# Antler stem cell exosomes alleviate pulmonary fibrosis via inhibiting recruitment of monocyte macrophage, rather than polarization of M2 macrophages in mice

**DOI:** 10.1038/s41420-023-01659-9

**Published:** 2023-09-28

**Authors:** Guokun Zhang, Liyan Shi, Jiping Li, Shengnan Wang, Jing Ren, Dongxu Wang, Pengfei Hu, Yimin Wang, Chunyi Li

**Affiliations:** 1 Institute of Antler Science and Product Technology, Changchun Sci-Tech University, 130600 Changchun, China; 2 China-Japan Union Hospital, Jilin University, 130033 Changchun, China; 3 College of Chinese Medicinal Materials, Jilin Agricultural University, 130118 Changchun, China

**Keywords:** Mesenchymal stem cells, Stem-cell research

## Abstract

Pulmonary fibrosis (PF), a chronic interstitial lung disease, is characterized by over-abundant deposition of extracellular matrix consisting mainly of collagen I. In previous studies, we demonstrated that deer antler stem cells (AnSCs), a novel type of adult stem cell, are capable of significantly down-regulating collagen formation in different organs and tissues and speculated that they could effectively treat PF *via* reducing collagen deposition in the lung tissue. In the present study, we found that administration of AnSCs improved the survival rate of PF mice and reduced lung fibrosis, collagen deposition and myofibroblast differentiation. The effects of AnSC treatment were significantly better than the positive control (adipose-derived stem cells). Interestingly, AnSC-Exos were almost equally effective as AnSCs in treating PF, suggesting that the effects of AnSCs on reduction of PF may be mainly through a paracrine mechanism. Further, AnSC-Exos reduced the number of M2 macrophages, a type of macrophage that secrets pro-fibrotic factors to accelerate fibrotic progression, in the lung tissues. In vitro experiments showed that the effects of AnSC-Exos on macrophage modulation were likely achieved *via* inhibition of the recruitment of circulating monocyte-derived macrophages (reducing the number of macrophages), rather than *via* inhibition of M2 polarization of macrophages. Inhibition of macrophage recruitment by AnSCs may be achieved indirectly via inhibiting CCL7 expression in fibroblasts; both let-7b and let-7a were highly enriched in AnSC-Exos and may play a critical role in the inhibition of CCL7 expression of fibroblasts. Collectively, the use of antler stem cells or their exosomes opens up a novel strategy for PF treatment in the clinical setting.

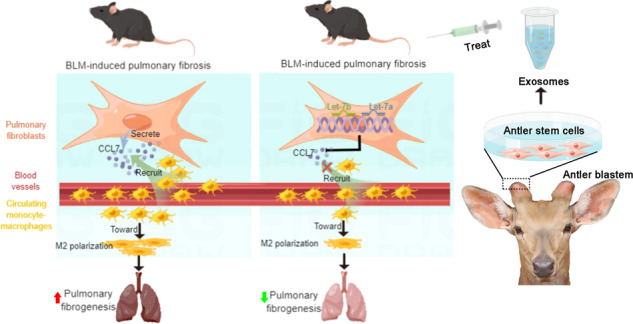

## Introduction

Pulmonary fibrosis (PF) is an end-point interstitial lung disease characterized by excessive deposition of extracellular matrix (ECM) consisting mainly of collagen I in the alveolar septa, causing septal interstitial thickening and respiratory disturbances [1–3]. Current therapies for PF are not satisfactory and more effective treatments are urgently needed, particularly given the current COVID-19 pandemic. The pathogenesis of PF presents as persistent microinjury of the alveoli, resulting in disruption of the alveolar architecture and macrophage migration and polarization [4, 5]. Classically-activated macrophages produce antimicrobial mediators, but macrophage responses can switch from a pro-inflammatory phenotype (M1) to an alternately activated state that exhibits an anti-inflammatory phenotype (M2). If this situation persists, M2 macrophages would secrete excessive pro-fibrotic factors such as transforming growth factor-β1 (TGF-β1) to induce fibroblast to myofibroblast transition (FMT), thereby triggering the pathological fibrosis pathway [6–8]. Myofibroblasts are known as the primary effector cells leading to fibrosis. Injury to the alveolar epithelium induces M2 macrophages to secrete TGF-β1, which triggers fibroblasts to express α-smooth muscle actin (α-SMA) and collagen I, and to stimulate FMT through the TGF-β/Smad signaling pathway [1]. Therefore, therapeutic approaches, either by repressing TGF-β1 signaling or repressing polarization to M2 macrophages could be expected to effectively alleviate the symptoms of PF [9].

In recent years, mesenchymal stem cell (MSC) transplantation has become a promising strategy for repairing tissue and organ injuries [10–12], due to their ability of homing to the injury site, low immunogenicity and differentiation into specific cell types required for tissue repair [11, 13–15]. In this respect, there is evidence that the lung is the first organ where intravenously-administered MSCs lodge, rendering MSCs a strong candidate for treatment of lung injury [16–19]. However, either MSC or embryonic stem cell (ESC) has its limitations such as potency or ethical concern [20], respectively. Therefore, an alternative type of stem cell with greater potency and fewer ethical concerns are needed.

Deer antler stem cells (AnSCs) have emerged as a novel type of adult MSCs. Antlers are the only mammalian organ that can fully regenerate (from the permanent bony protuberances or pedicles [21, 22]), with the first step in antler regeneration being the scarless wound healing over the pedicle stump following the casting of the previous hard antler [23]. This wound healing depends on the AnSCs in the adjacent pedicle periosteum or the earliest antler blastema [24, 25]. This ability of AnSCs to promote scarless wound healing can also be realized in other species, such as rats [14]. These therapeutic effects of AnSCs have pointed to their potent anti-fibrotic activities. Therefore, we considered the possibility that AnSCs might have therapeutic potential in treatment of organ fibrosis, and our recent study confirmed the effectiveness of AnSC treatment in the alleviation of liver fibrosis [26]. In this study, we elucidated whether AnSCs have the potential as a novel MSC source for treatment of PF.

Studies on the therapeutic effects of transplanted MSCs revealed that only few MSCs survive at the injury site and differentiate into cell types required for tissue repair, and that the effects of MSCs are achieved mainly through a paracrine mechanism including secretion of soluble factors and exosomes. In this respect, exosomes can deliver functional RNAs, DNAs, and protein factors to recipient cells in the tissue to be repaired.

The present study sought to investigate the antifibrotic effects of AnSCs using a bleomycin (BLM)-induced mouse model of PF and determined whether the antifibrotic effects were achieved mainly through a paracrine pathway by including a exosome (AnSC-Exos) treatment. We found that AnSC effectively alleviated symptoms of PF and the outcomes were significantly better than that of adipose-derived stem cells (ADSCs). Notably, AnSC-Exos achieved comparable effects to the AnSCs, suggesting that the antifibrotic function of AnSCs is likely realized *via* a paracrine pathway, mediated via products of exosomes. Further mechanistic studies showed that the AnSC-Exos-specific miRNAs, let-7b and let-7a, appear to have played a critical role in inhibiting CCL7 expression of the fibroblasts which, in turn, reduced recruitment of monocyte-derived macrophages on site and macrophage polarization to M2, contributing to the alleviation of PF. Overall, we believe that our study has opened up a new avenue for using AnSC-Exos to effectively treat PF in the clinic setting.

## Results

### AnSCs alleviated pulmonary fibrosis in the BLM-induced PF mice *via* a paracrine mechanism

**Fig. 1:**
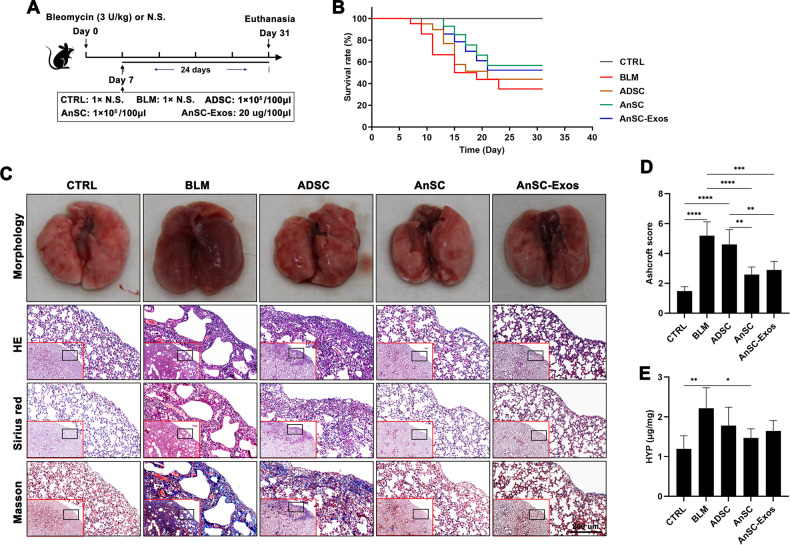
AnSCs and AnSC-Exos alleviated pulmonary fibrosis in the BLM-induced PF in mice. **A** Schematic showing experimental design. **B** Survival rate of the model mice. **C** Morphology and histology of the lung tissue with different stains (HE, Sirius red and Masson); scale bar = 200 μm. **D** Ashcroft score of histological images (scored by three pathologists blind to treatment). **E** HYP content of the lung tissue. Value: Mean ± SEM; *p < 0.05, **p < 0.01, ***p < 0.001, ****p < 0.0001 as indicated by Student’s *t* test; *n* = 5. ADSC adipose-derived stem cells, BLM bleomycin, N.S. normal saline, CTRL control, HE hematoxylin and eosin, HYP hydroxyproline.

We first treated the mice with AnSCs or AnSC-Exos through intravenous injection on day 7 after BLM induction to evaluate the effect on PF (Fig. 1A). Compared with the normal saline group, control mice treated with BLM (model mice) had the lowest survival rate (35%); treatments with AnSCs (57%) and AnSC-Exos (52%) significantly increased the survival rate (Fig. 1B). Both the degree of lung tissue damage (Ashcroft score) and collagen deposition (histology) were also significantly reduced in both the AnSC and AnSC-Exos treatment groups compared to the normal saline group (Fig. 1C, D; *p* < 0.0001, *p* < 0.001). Besides, hydroxyproline (HYP), a marker of collagen, was also decreased significantly after treatments with either AnSCs or AnSC-Exos (Fig. 1E; *p* < 0.05). Overall, the therapeutic effects of both AnSCs and AnSC-exos on PF were comparable to each other, but were greater than that of ADSC treatment.

The expression levels of both collagen I and collagen III in the lung tissues were measured *via* immunofluorescence (IF) staining, with both collagen I and collagen III highly expressed in the lung tissues of the model mice; expression levels were significantly lower in the AnSCs (*p* < 0.0001, *p* < 0.01) or AnSC-Exos (*p* < 0.0001, *p* < 0.01) group, but there was no significant difference between these two groups (Fig. S2A–C). Moreover, the effects of AnSCs on reduction of the collagen I expression was significantly higher than that of ADSC (*p* < 0.01). The results of both western blot and qRT-PCR further supported the IF results; that is, treatment with both AnSCs and AnSC-Exos significantly reduced the expression levels of collagen I and collagen III in the lung tissue, particularly collagen I (Fig. S2D–H). Overall, the evidence points to the fact that the effects of AnSCs on PF in the model mice were highly likely to have been mediated via a paracrine pathway per the exosomes.

### AnSCs and AnSC-Exos reduced myofibroblast differentiation and the number of M2 macrophages

**Fig. 2:**
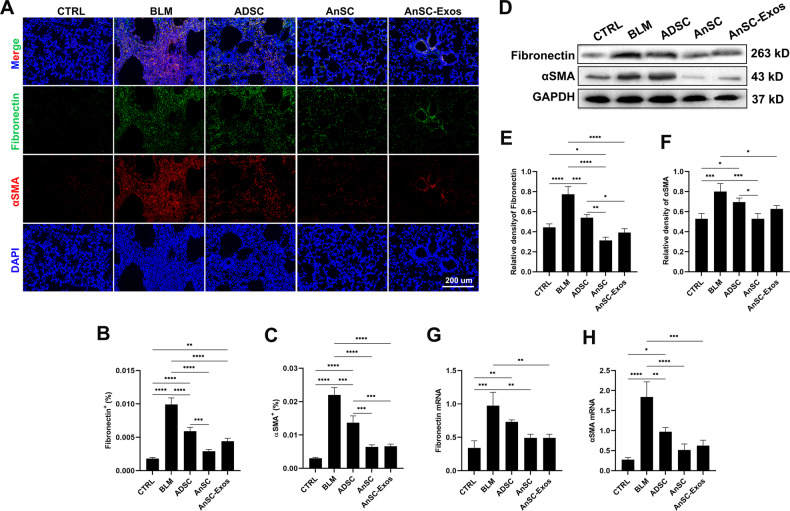
Inhibition of myofibroblast differentiation by AnSCs and AnSC-Exos in the lung tissue of BLM-induced PF in mice. **A** IF stains of fibronectin and α-SMA; scale bar = 200 μm. **B**, **C** Percentages of fibronectin^+^ cells and α-SMA^+^ cells, respectively. **D**–**F** Western blot bands and relative intensities of fibronectin and α-SMA, respectively. **G**, **H** Relative mRNA levels of fibronectin and α-SMA, respectively. Note that the lowest values in all detected parameters were found in the treatments of AnSCs and AnSC-Exos. Value: Mean ± SEM; *p < 0.05, **p < 0.01, ***p < 0.001, ****p < 0.0001 as indicated by Student’s *t* test; *n* = 3. αSMA, α smooth muscle actin.

We sought to determine whether the high abundance of collagens in the lung tissues of the model mice resulted from an excess of FMT. The classic markers for myofibroblasts are α-SMA and fibronectin [27, 28], and both markers were highly expressed in the lung tissue of the model mice. The expression levels (IF staining) of α-SMA and fibronectin (*p* < 0.0001) were significantly reduced in the AnSCs or AnSC-Exos groups but there was no significant difference between these two treatments (Fig. 2A–C). The reduction in expression levels of fibronectin and α-SMA in the AnSCs and AnSC-Exos were significantly stronger than those of ADSCs (*p* < 0.01). The results of both western blot and qRT-PCR further support the findings from the IF staining (Fig. 2D–H). This indicates that the high abundance of collagens was caused by excessive FMT.

**Fig. 3:**
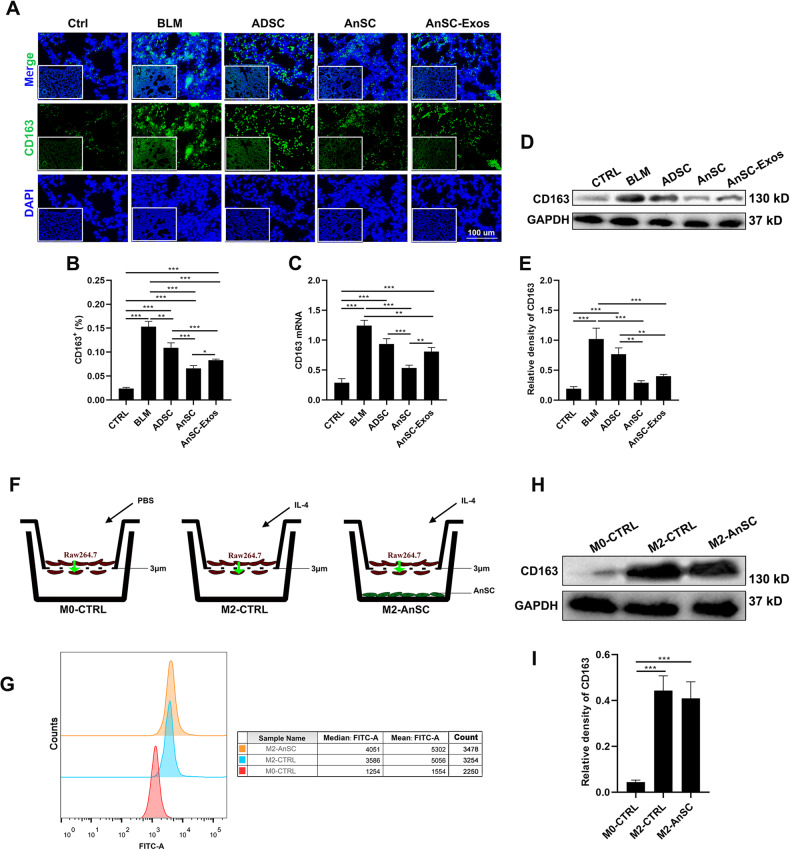
Numbers of M2 macrophages under treatment with AnSCs and AnSC-Exos in the BLM-induced PF mice. **A** IF staining of CD163 on the lung tissue sections; scale bar = 100 μm. **B** Percentages of CD163^+^ cells. **C** Relative mRNA level of CD163. **D**, **E** Western blot bands and the relative intensities of CD163. **F** Schematic showing in vitro experimental design: Raw264.7 cells were cultured in the inserts in all groups, AnSCs cultured in the wells of the third group, and IL4 was added to the wells of the second and third groups to induce macrophage polarization toward M2 (the green arrow points the direction of cell migration). **G**–**I** Expression levels of CD163 using flow cytometry and western blot analysis, respectively. Note: AnSC-Exos effectively reduced the number of M2 macrophages in the BLM-induced PF mice in vivo, but had no significant effect on M2 polarization in vitro, indicating that the in vivo effect was achieved indirectly. Value: Mean ± SEM; *p < 0.05, **p < 0.01, ***p < 0.001 as indicated by Student’s *t* test; *n* = 3.

It is reported that TGF-β1, the pro-fibrotic factor, is the potent trigger for FMT, and TGF-β1 is mainly secreted by M2 macrophages [29]. Thus, we sought to test whether the excessive FMT was caused by polarization of macrophages toward M2. Our results showed that CD163, the marker gene of M2 macrophages [30], was highly expressed in the lung tissues of the model mice *via* IF staining, western blot analysis and qRT-PCR analysis (Fig. 3A–E). Treatment with either AnSCs or AnSC-Exos reduced significantly the expression levels of CD163 (Fig. 3A–E). Also, the effects of both AnSC and AnSC-exos treatments were comparable to each other but were significantly stronger than that of ADSC treatment. These results suggest that AnSCs/AnSC-Exos can effectively reduce FMT possibly *via* reducing numbers of M2 macrophages in the lung tissue.

### AnSCs and AnSC-Exos reduced M2 macrophages *via* inhibiting recruitment of monocyte-derived macrophages

To reveal the mechanism underlying the reduction in number of M2 macrophages by treatments with AnSCs and AnSC-Exos, we took an in vitro co-culture approach between AnSCs and Raw264.7 macrophages; IL-4 was added in the co-culture to induce M2 polarization of Raw264.7 cells (Fig. 3F). Results showed that AnSCs in the co-culture system failed to prevent IL-4 induction of M2 polarization, evidenced by the expression level of CD163 (M2 marker gene) using flow cytometry (FCM) and western blot analysis (Fig. 3G–I).

**Fig. 4:**
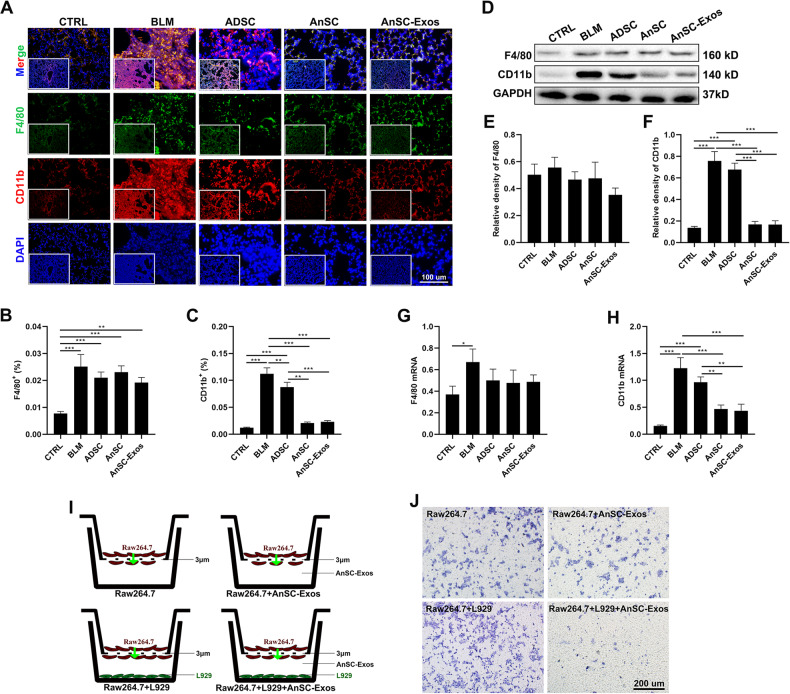
Inhibition of the recruitment of blood-derived macrophages by AnSCs and AnSC-Exos in the lung tissues of BLM-induced PF mice. **A** IF stains of F4/80 (marker for resident macrophages) and CD11b (marker for blood-derived macrophages) in lung tissue sections. **B**, **C** Percentages of F4/80^+^ cells and CD11b^+^ cells, respectively. **D**–**F** Western blot bands and the relative intensities of F4/80 and CD11b, respectively. **G**, **H** Relative mRNA levels of F4/80 and CD11b, respectively. Note that both AnSCs and AnSC-Exos effectively inhibited recruitment of blood-derived macrophages but had no significant effect on the number of resident macrophages. **I** Schematic showing in vitro experimental design: Raw264.7 cells were cultured in the inserts in all groups (without IL4), L929 fibroblasts were cultured in the wells of the third and fourth groups, and AnSC-Exos was added to the wells in the second and fourth groups to induce migration of Raw264.7 cells (the green arrow points the direction of cell migration). **J** Crystal violet staining of the migrated Raw264.7 cells; scale bar = 200 μm. Note that AnSC-Exos indirectly inhibit the recruitment of macrophages by fibroblasts. Mean ± SEM; *p < 0.05, **p < 0.01, ***p < 0.001 as indicated by Student’s *t* test; *n* = 3.

Next, we set to determine the origin of macrophages (lung-resident or monocyte-derived) in the lung tissue of the model mice using macrophage specific markers F4/80 (lung-resident) and CD11b (monocyte-derived) [31, 32]. Results showed that expression levels of both F4/80 and CD11b were increased significantly in the lung tissues of the model mice; further, treatment with AnSCs or AnSC-Exos significantly decreased the expression level of CD11b (*p* < 0.001; Fig. 4A, C), but did not alter the expression level of F4/80 (Fig. 4A, B). The results of both western blot and qRT-PCR analysis confirmed the staining results (Fig. 4D–H).

Therefore, the effects of AnSCs on reduction of M2 macrophages may be achieved mainly through inhibition of recruitment of circulating monocyte-derived macrophages to the lesion, and in so doing, the overall number of macrophages is decreased.

### AnSC-Exos inhibited macrophage recruitment through targeting CCL7 expression of fibroblasts

Next, we carried out experiments to determine whether AnSC-Exos could directly inhibit migration of macrophages in vitro; in this model, positive results would support the finding that AnSC-Exos inhibited recruitment of monocyte-derived macrophages in vivo. Unexpectedly, we failed to detect significant effects of AnSC-Exos on the migration of Raw264.7 cells in a cell migration assay (Fig. 4I and J). The results indicate that effects of AnSC-Exos on inhibition of recruitment of monocyte-derived macrophages may be through an indirect pathway in vivo.

It is known that fibroblasts secrete chemokines to attract circulating macrophages to the lesion [33, 34], and hence it is possible that AnSC-Exos inhibited recruitment of circulating macrophages through targeting fibroblasts. To test this hypothesis, we set up a co-culture system between Raw264.7 macrophages and L929 fibroblasts. The results showed that addition of AnSC-Exos to the co-culture system significantly inhibited L929-induced migration of Raw264.7 macrophages (Fig. 4J). These results confirmed the involvement of fibroblasts in this transduction loop.

CCL7 is known as a fibroblast-secreted chemokine and that it is critical to macrophage recruitment [35–37]. We found that the CCL7 was highly expressed in the lung tissues of the model mice (Fig. S3), and that treatment with either AnSCs or AnSC-Exos reduced significantly the expression level of CCL7 (Fig. S3). Addition of CCL7 in the culture system induced significantly the migration of Raw264.7 cells in a dose-dependent manner (Fig. S4). Furthermore, we found that AnSC-Exos decreased significantly the expression level of CCL7 of the L929 fibroblasts (*p* < 0.001; Fig. S5).

### Suppression of CCL7 expression by AnSC-Exos may be via their cargo-miRNAs, particularly let-7b and let-7a

The effects of exosomes depend critically on their contents and miRNA is one of the most important cargos involved in regulating recipient cells [38–40]. Therefore, we sought to determine whether miRNAs were the critical players in down-regulation of CCL7 expression of fibroblasts. Sequencing of AnSC-Exos identified multiple miRNAs, with the 20 most abundant being let-7b, let-7a, miR-21, let-7c, let-7i, let-7g, let-7e, miR-423-5p, miR-100, let-7f, miR-184, miR-143, miR-26a, miR-151-5p, miR-99a-5p, miR-486, miR-99b, miR-92a, miR-126-5p, and miR-27b (Fig. S6A). The potential target genes of let-7b and let-7a, the two most abundant in AnSC-Exos, were predicted through bioinformatics analysis (TargetScan, http://www.targetscan.org/); these two miRNAs directly target CCL7 (Fig. S6B). Subsequently, the mimics and inhibitors of let-7b and let-7a were used to experimentally assess their functions *via* cell migration assays.

**Fig. 5:**
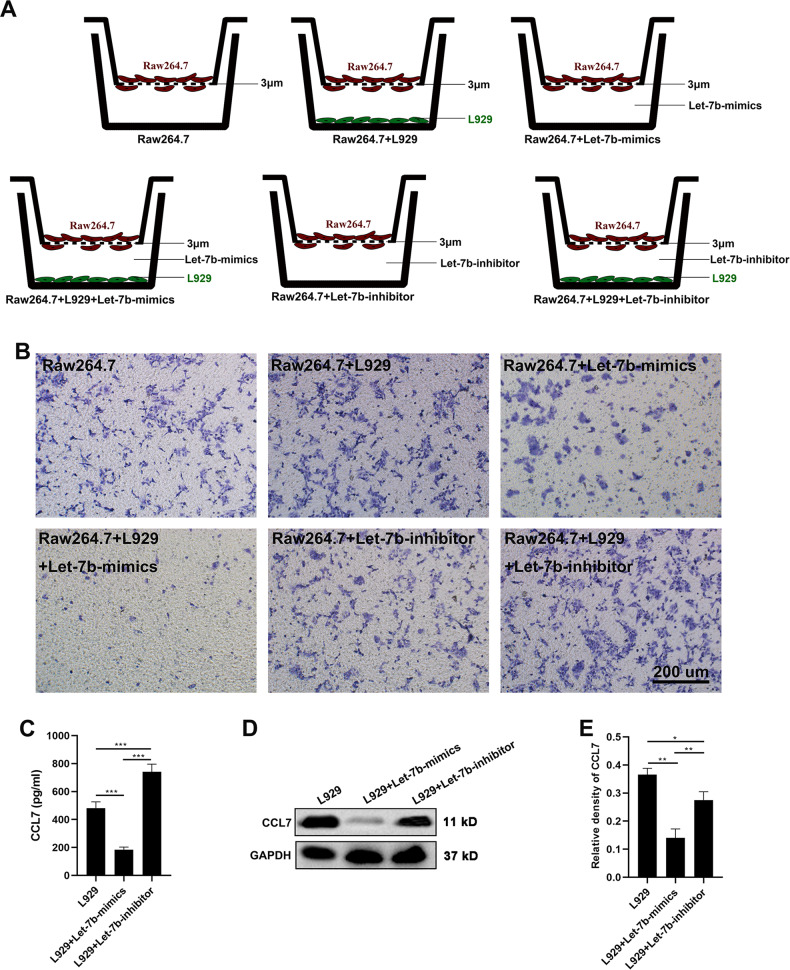
Inhibition of fibroblast-induced macrophage migration by AnSC-Exos-let-7b. **A** Schematic showing experimental design: Raw264.7 cells were cultured in the inserts of all groups, L929 fibroblasts cultured in the wells of the second, fourth and sixth; let-7b-mimics were added in the wells of the third and fourth groups, and let-7b-inhibitor added in fifth and sixth groups. **B** The migrated Raw264.7 cells were stained with crystal violet; scale bar = 200 μm. Note that let-7b itself had no significant effect on the migration of Raw264.7 cells but significantly inhibited the L929-induced migration of Raw264.7. **C**–**E** Expression levels of CCL7 using ELISA (supernatant) and western blot (L929 cells). Value: Mean ± SEM; *p < 0.05, **p < 0.01, ***p < 0.001 as indicated by Student’s *t* test; *n* = 3.

Firstly, mimics or inhibitors of let-7b were added to the co-culture system between Raw264.7 and L929 to determine whether let-7b can functionally affect chemotaxis to RAW 264.7 cells (Fig. 5A). While let-7b (mimics or inhibitors) had no direct effect on the migration of Raw264.7, mimics of let-7b inhibited L929-induced migration of Raw264.7 cells; inhibitors of let-7b promoted L929-induced migration of Raw264.7 cells (Fig. 5B). In addition, the effects of let-7b on the chemotaxis of fibroblasts for macrophages might be achieved through regulating the expression levels of CCL7 of fibroblasts, evidenced by the inhibitory action of let-7b on CCL7 expression of L929 cells detected via ELISA and western blot (Fig. 5C–E).

**Fig. 6:**
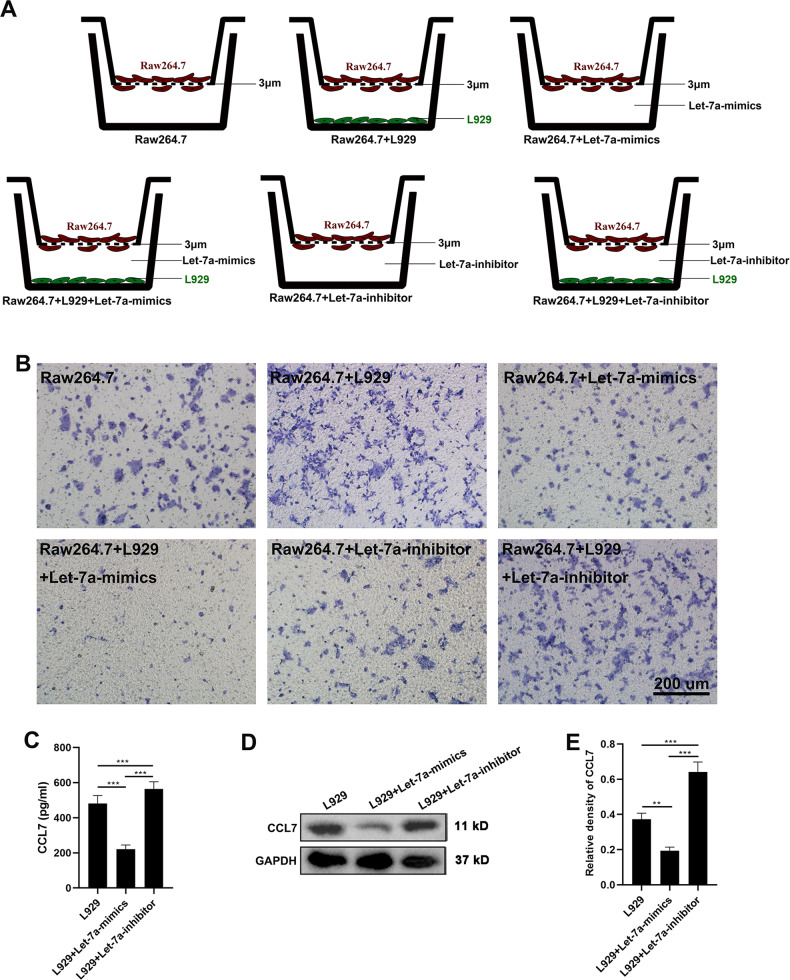
Inhibition of fibroblast-induced macrophage migration by AnSC-Exos-let-7a. **A** Schematic showing experimental design (same as Fig. 5). **B** The migrated Raw264.7 cells were stained with crystal violet. Note that let-7a itself had no significant effect on migration of Raw264.7 cells but did inhibit the L929-induced migration of Raw264.7; scale bar = 200 μm. **C**–**E** Expression levels of CCL7 using ELISA (supernatant) and western blot (L929 cells). Value: Mean ± SEM; **p < 0.01, ***p < 0.001 as indicated by Student’s *t* test; *n* = 3.

To assess the role of let-7a in AnSC-Exos, mimics or inhibitors of let-7a were added to the co-culture system between Raw264.7 and L929 to determine whether let-7a can affect the chemotaxis of RAW 264.7 cells (Fig. 6A). Similar to let-7b, let-7a inhibited significantly the L929-induced migration of Raw264.7 (Fig. 6B). Further investigation also showed that the effect of let-7a on the chemotaxis of fibroblasts for macrophages might be achieved by regulating the expression levels of CCL7 of fibroblasts, evidenced by let-7a inhibiting CCL7 expression of L929 cells detected via ELISA and western blot (Fig. 6C–E). Overall, our results suggest that AnSC-Exos could effectively inhibit macrophage recruitment at least partially through inhibition of let-7b and let-7a on CCL7 expression of the fibroblasts.

## Discussion

We have shown in the study that treatments using AnSCs or AnSC-Exos were equally effective in alleviating the symptoms of PF in a mouse model system and increasing survival rate of affected mice. The effectiveness of exosomes shows that the effects of AnSCs were likely effected through a paracrine mechanism. AnSC-Exos reduced significantly the number of M2 macrophages possibly via inhibition of recruitment of circulating monocyte-macrophages, rather than through M2 polarization. Further in vitro studies found that inhibition of macrophage recruitment by AnSC-Exos is likely indirect through suppression of CCL7 expression in fibroblasts by AnSC-Exos-cargo-miRNAs, let-7a and let-7b. The present study provides an alternative option for investigation as a treatment of PF in a clinical setting.

Recent studies have convincingly demonstrated the effects of MSCs on the reduction of PF, but MSCs from different sources exhibit different potencies in vitro and in vivo [16–19, 41]. In this study, we found that AnSCs had more potent mitogenic effects in vitro and effects on animal survival and reduction of PF symptoms in the mouse model in vivo than other types of MSCs, probably because AnSCs possess partial ESC properties [21, 42, 43]. Consistently, we have found highly potent anti-fibrotic effects of AnSCs in previous studies, including regenerative wound healing (reduction of scaring) and reduction of liver fibrogenesis [14, 26, 44].

The role of MSCs in repair of tissue injury, including immunomodulation, anti-apoptotic activity, pro-angiogenesis, has been shown to be through a paracrine mechanism, effected via secreted exosomes [12, 45, 46]. In the present study we found (after intravenous injection of CFDA-SE-labeled AnSCs into the PF model mice) that the majority of AnSCs were detected within 1–7 days but only few AnSCs survived to day 14 (Fig. S7), indicating that it was not the AnSCs per se that participated in the tissue repair, but paracrine factors that executed the main therapeutic effects. Exosomes of MSCs contain cell-specific “cargos” of mRNAs, DNAs, proteins, and lipids, and these cargo-components can effectively modulate the activities of recipient cells and play essential roles in the repair of tissue injury [39, 47]. Compared to cells per se, exosomes have advantages in storage and transport, and have been shown to pose a lesser risk of tumor generation or immunogenicity [39, 48]. Therefore, AnSC-exos may have greater potential than AnSCs as a novel cell-free therapeutic for PF treatment.

Initiation of PF has been attributed to the persistent and excessive activation of myofibroblasts, as the latter is the main cell type for ECM deposition [1–3]. Injury of lung alveolar structure triggers mediators of M2-macrophage-derived (e.g., TGF-β1) to promote FMT through the TGF-β/Smad signaling pathway [1, 8]. Therefore, in theory, repression of M2 polarization would reduce FMT, and in turn inhibit the development of PF. Indeed, Moroncini et al. (2018) found that umbilical cord-derived MSCs (uMSCs) significantly reduced PF by inhibiting M2 polarization [49], in the present study, although AnSC-Exos alleviated PF symptoms in the mouse model through a reduction in the number of M2 macrophages and thus suppressed FMT, the way of reduction in the numbers of M2 macrophages was different to that reported for uMSCs by Moroncini *et al* (2018), namely suppression of M2 polarization. In our study, AnSC-Exos clearly failed to suppress polarization of M2 macrophages, but did inhibit recruitment of circulating monocyte-macrophages. In so doing, the total number and the number of M2 macrophages of macrophages were reduced.

It is well known that circulating macrophages are mainly attracted to lesions by chemokines secreted by fibroblasts [33, 34]. Our in vitro experiments were designed to determine whether AnSC-Exos inhibited macrophage recruitment directly, or indirectly *via* fibroblasts. We found that AnSC-Exos themselves failed to influence migration of macrophages whereas, when fibroblasts were added/co-cultured with macrophages, AnSC-Exos significantly inhibited migration of the macrophages (Fig. 4J), clearly indicating that the AnSC-Exos played their role in suppressing recruitment of macrophages indirectly *via* fibroblasts. To our knowledge this is the first indication of such an indirect effect of MSCs through recruitment of macrophages, thus our study may have identified a new target for the treatment of PF.

The mechanism by which AnSC-Exos inhibit the secretion of chemokines by the fibroblasts is unknown. However, we found that CCL7 was highly expressed in the lung tissue of the PF mice, and significantly induced macrophage migration in vitro in a dose-dependent manner. CCL7 is a chemokine and potent monocyte-macrophage attractant, normally expressed at low levels in fibroblasts and upregulated by a range of stimuli, including viruses and interferons [37]; for example, CCL7 is highly expressed in pulmonary fibroblast lines from patients with interstitial pneumonia [37]. Therefore, it seems likely that AnSC-Exos suppressed CCL7 expression in the fibroblasts, resulting in suppression of macrophage migration.

It is known that miRNAs are the most important effective substances of exosomes [39]. In our study, we identified the 20 most abundant miRNAs in the AnSC-Exos (Fig. S6A). Interestingly, the most abundant miRNAs, let-7a and let-7b, are reported to directly target CCL7 (Fig. S6B), and our functional analysis in the present study confirmed that these two miRNAs could inhibit expression of CCL7 of the fibroblasts. While the actual mechanism by which CCL7 promotes initiation of PF has not been reported, studies have shown that both lung alveolar epithelial cells and fibroblasts can produce CCL2 and CCL12, which can attract macrophages to the site, thereby promoting the development of PF [50, 51]. Understanding the molecular mechanism underlying the effects of AnSC-Exos on PF would facilitate development of a novel strategy for the effective treatment of this debilitating disease.

**Fig. 7:**
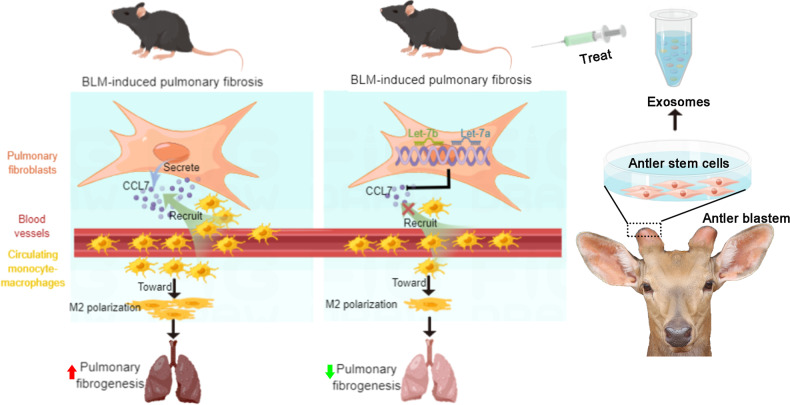
The miRNAs l (et-7b, let-7a) of AnSC-Exos-origin significantly reduced CCL7 expression of fibroblasts, inhibited recruitment of monocyte-macrophages, leading to a reduction in the accumulation of M2 macrophages, contributing to the alleviation of PF.

In conclusion, the therapeutic effects of AnSCs on PF were found to be through their secreted exosomes. The miRNAs, let-7b and let-7a, of AnSC-Exos-origin reduced CCL7 expression by fibroblasts, inhibited recruitment of monocyte-macrophages, leading to a reduction in M2 macrophage accumulation, and contributed to the alleviation of PF (Fig. 7). Therefore, the antler stem cell appears to have potential as an alternative source of exosome for stem cell therapy, and AnSC-Exos have potential as a novel cell-free therapeutic for the treatment of PF in clinical settings.

## Materials and methods

### Cell culture

AnSCs were isolated from the tissue of antler blastema (3–7 days after hard antler button casting) using the methods reported by Li et al. [23, 42]. Briefly, skin covering the antler blastema was cut open to expose the underneath blastema tissue, which was then removed and cut into 0.2 mm in cubes, washed three times with PBS, and digested in type I collagenase for 30 min (37 °C). The digested tissue was transferred to a 10 cm culture dish for primary cell culture. The cells were sub-cultured when they reached confluence, and used in subsequent experiments (2–5 passages).

**Fig. 8:**
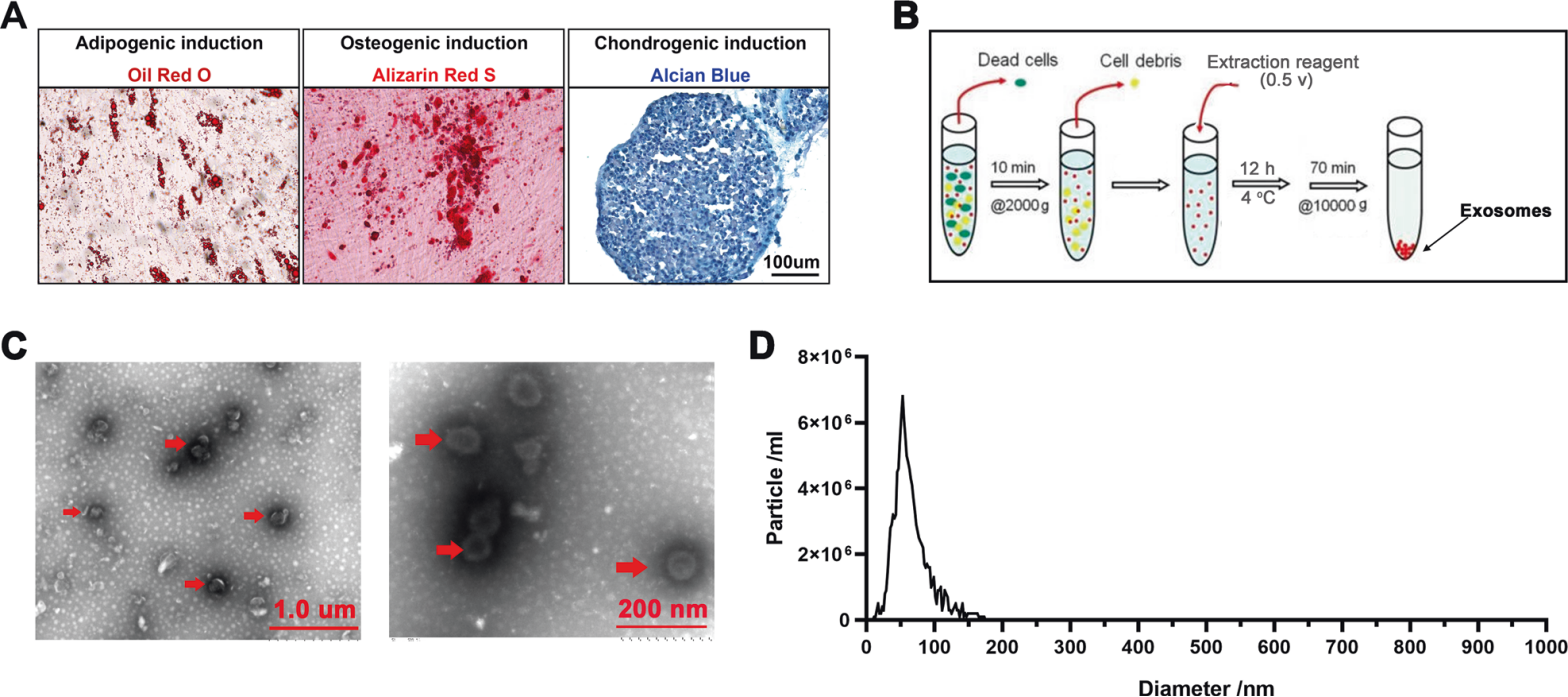
Preparation and characterization of AnSCs and AnSC-Exosomes. **A** Oil Red O, Alizarin Red S or Alcian Blue staining to show the differentiation of AnSCs to adipocytes, osteocytes or chondrocytes, respectively. **B** Schematic representation of AnSC-Exos preparation procedure. **C** Morphology of AnSC-Exos under transmission electron microscopy; scale bar = 1.0 μm or 200 nm. **D** Particle size distribution of AnSC-Exos using NanoSight. AnSCs, Antler stem cells; AnSC-Exos, AnSC-derived exosomes.

AnSCs were characterized by using IF staining for mesenchymal stem cell markers, including CD73, CD90, Nestin, and Sox2 (Fig. S1), as previously described [21, 42]. When the density of AnSCs in the culture dish reached 70%, the medium was decanted, washed with PBS, fixed in methanol for 30 min, and then incubated with the specific primary antibody. The procedures for all antibodies (Table S1) were identical, except for Nestin and Sox2 staining where they were pre-permeabilized with Triton X-100 for 10 min. After visualization of nuclei with DAPI (Beyotime, China), images were viewed under a fluorescence microscope (EVOS M5000, USA). The multipotency of AnSCs was detected *via* induction of osteogenic (Alizarin Red S staining), chondrogenic (Alcian Blue staining), and adipogenic (Oil Red O staining) differentiation (Fig. 8A).

Mouse ADSCs, L929 fibroblast line, and Raw264.7 macrophage line were cryopreserved in our laboratory. The cells (AnSCs, ADSCs, L929, Raw264.7), were cultured in DMEM (Gibco, USA) containing 10% fetal bovine serum (FBS; Gibco, USA) supplemented with 1% penicillin/streptomycin (BI, Israel) at 37 °C, 5% CO_2_, saturated humidity.

### Exosome isolation

AnSCs were cultured in a DMEM medium containing 10% FBS, and when density reached 80%, the medium was replaced with UltraCULTURE serum-free medium (Lonza, USA) containing 1% of 200 mM L-glutamine for 48 h. The conditioned medium (after removal of dead cells and cell debris) was collected for exosome isolation using the Hieff® Quick exosome isolation kit (Yeasen, China). Specifically, 0.5 volume of extraction reagent was mixed with the conditioned medium, held at 4 °C for 12 h, and then centrifuged at 10,000 *g* for 70 min to collect exosomes (precipitation, Fig. 8B). The prepared AnSC-exos were stored at −80 °C for use. AnSC-Exos (Fig. 8C, D) were characterized using transmission electron microscopy and NanoSight NS300 (Malvern Instruments, UK) [39].

### Mouse model and treatments

75 C57BL/6 mice (male, 6–8 weeks old; Changsheng, China) were randomly divided into 5 groups for the follow-up experiments after 1 week of laboratory acclimatization. All mouse experiments were approved by the Animal Ethics Committee of Changchun Sci-Tech University (Approval No.: CKARI202001). Sample size was calculated, based on the ethics committee instructions, by the aid of http://www.biomath.info/power/prt.htm. The study’s primary outcome was improving survival rate and reducing pulmonary fibrosis, and 15 rats/group were needed based on the power calculation. No animal was excluded during the experiment. The work has been reported in accordance with the ARRIVE guidelines (Animals in Research: Reporting In Vivo Experiments). Mice were intratracheally treated with BLM (dose of 3 U/kg) on day 0 to induce PF. The control group received similar treatment, but with normal saline (N.S.) instead of BLM. BLM-induced mice were received the following treatment: ADSC (1 × 10^5^), AnSC (1 × 10^5^), AnSC-Exos (20 μg) in 100 μL of N.S., and an equal volume of N.S. Treatments were carried out *via* intravenous injection on day 7, and all mice were euthanized on day 31 for tissue collection. The mice in the control group were treated with 100 μL of N.S. The lung tissues were collected, one part was fixed in 10% formaldehyde solution for subsequent histological examination, and the other was stored at −80 °C for subsequent molecular biology evaluation.

### HYP detection

The HYP content of the lung homogenate was detected using an HYP assay kit (Solarbio, China) according to the manufacturer’s instruction. The absorbance of the samples was measured at 550 nm.

### Histology

Lung tissue was fixed in 10% formaldehyde solution for 48 h and rinsed with water. Following sequential dehydration in gradient ethanol and xylene, the tissue was embedded in paraffin and cut into 5.0 μm thick sections for subsequent staining. The sections were stained with hematoxylin-eosin (HE), Sirius red and Masson according to the manufacturer’s instructions (Solarbio, China) and photographed using a Microscope (Precipoint M8, Germany). Ashcroft score was used to evaluate the development of pulmonary lesions according to the histological images, which was determined by three pathologists blind to the study design.

IF staining was performed on the paraffin sections using specific primary antibodies: collagen I, collagen III, α-SMA, Fibronectin, CD163, F4/80, CD11b, and CCL7. The sections were, thereafter, stained with Cy3- or AF488-labeled secondary antibodies. After visualization of nuclei with DAPI (Beyotime, China), images were viewed under a fluorescence microscope (EVOS M5000, USA). The numbers of the positively expressed (^+^) cells and total cells per 20 × high-power field were quantified using Image-Pro Plus software, and the percentage of positive (^+^) cells to total cells was calculated.

### Treatment of the cultured cells and co-culture

1. Co-culture of Raw264.7 macrophages with AnSCs (To detect the effect of AnSCs on the polarization of Raw264.7 toward M2).
  Raw264.7 was seeded in the transwell insert of a 6-well plate with a density of 1 × 10^5^ cells/ml; the medium was added with IL-4 (5 ng/ml) to induce M2 polarization. AnSCs were seeded in the 6-well plates at a density of 1 × 10^5^ cells/ml (2 ml/well) for 48 h culture. To establish the co-culture between IL-4-induced Raw264.7 and AnSCs, we transferred each culture insert containing IL-4 pretreated Raw264.7 cells to each well that had been cultured with AnSCs (transwell chamber). After 12 h of co-culture, cells in the inserts were collected for FCM assay and western blot analysis to detect CD163 expression, a marker for M2 polarized macrophages.
2. Co-culture of Raw264.7 macrophages with L929 fibroblasts (Treated by AnSC-Exos; to detect the effect of AnSCs on the migration of Raw264.7).
  Raw264.7 was seeded in the transwell insert of a 24-well plate at a density of 1 × 10^5^ cells/ml. L929 was seeded in the wells at a density of 1 × 10^5^ cells/ml (2 ml/well) for 48 h culture; the medium was added with AnSC-Exos (50 ng/ml). To establish the co-culture between Raw264.7 and L292 cells, we transferred each culture insert containing Raw264.7 cells to each well that had been cultured with L292 cells (transwell chamber). After 12 h of co-culture, crystal violet staining was performed for Raw264.7 cells on the underside of the insert membrane.
3. Co-culture of Raw264.7 macrophages with L929 fibroblasts (Treated by AnSC-Exos-specific miRNAs).
  Raw264.7 was seeded in the transwell insert of a 24-well plate at a density of 1 × 10^5^ cells/ml. L929 was seeded in the wells at a density of 1 × 10^5^ cells/ml (2 ml/well) for 48 h culture; the medium was added with miRNA (Let-7b or Let-7a) mimics or inhibitor (50 nM). To establish the co-culture between Raw264.7 and L292 cells, we transferred each culture insert containing Raw264.7 cells to each well that had been cultured with L292 cells (transwell chamber). After 12 h of co-culture, crystal violet staining was performed for Raw264.7 on the underside of the insert membrane.
4. Raw264.7 macrophages cultured with CCL7 in the transwell system.
  Raw264.7 cells was seeded in the transwell insert of a 24-well plate with a density of 1 × 10^5^ cells/ml. Different concentrations of CCL7 (0, 2.5, 5, 10, 25, and 50 ng/ml) were added in the transwell chamber. After 12 h of co-culture, crystal violet staining was performed for Raw264.7 on the underside of the insert membrane.
5. Culture of L929 fibroblasts treated with AnSC-Exos or AnSC-Exos-specific miRNAs.

L929 was seeded in a 24-well plate with a density of 1 × 10^5^ cells/ml; the medium was added with AnSC-Exos (50 ng/ml) or AnSC-Exos-specific miRNA (Let-7b or Let-7a) mimics/inhibitor (50 nM) for 48 h of culture. The expression of CCL7 in L929 conditioned medium and in L929 was detected *via* ELISA and western blot, respectively.

### Western blot

Total protein was extracted from the cells or lung tissues using RIPA reagent (Invitrogen, USA). The proteins were then loaded and separated *via* polyacrylamide SDS gel. Following electrophoresis, protein bands were transferred onto polyvinylidene fluoride membranes (Millipore, MA), blocked in 5% (w/v) non-fat milk, and incubated with the primary antibodies overnight and the secondary antibody for 2 h. Finally, blots were incubated with ECL. Target protein bands were quantified using Image J software and normalized to the signal intensity of GAPDH.

### Quantitative real-time polymerase chain reaction (qRT-PCR)

Total RNA of the cells or lung tissues were isolated using Trizol reagent (Invitrogen, USA); cDNAs thereof were synthesized using cDNA Synthesis Kit (Takara, Japan). The qRT-PCR was performed to examine the expression levels of target RNAs using SYBR Green Master (Roche, Germany) in qTOWER 3 G (Analykit Jena AG, Germany). Results of mRNA quantification were normalized against GAPDH and calculated using the ^ΔΔ^Ct method. All reactions were performed in triplicate. The information on primers used is listed in Table S2.

### Statistical analysis

Data are expressed as means ± SEM (*n* ≥ 3). Statistical analysis was conducted using Graphpad Prism software with a one-way ANOVA; *p < 0.05, **p < 0.01, ***p < 0.001, or ****p < 0.0001 was considered statistically significant or highly significant.

### Supplementary information

SUPPLEMENTAL FIGURES AND TABLES

Original Data File

## Data Availability

All datasets generated and analyzed during this study are included in this published paper and its Supplementary Information files. Additional data are available from the corresponding author on reasonable request.
