# Supplementary material for: Antler stem cell exosomes alleviate pulmonary fibrosis via inhibiting recruitment of monocyte macrophage, rather than polarization of M2 macrophages in mice
Source: Cell Death Discov. 2023 Sep 28;9:359. doi: 10.1038/s41420-023-01659-9 (PMC10539297; doi:10.1038/s41420-023-01659-9)
Supplement: Supplementary file 1 — SUPPLEMENTAL FIGURES AND TABLES [file 41420_2023_1659_MOESM1_ESM.docx]

**Supplemental Materials**

**Table S1 Antibodies**

| **Antibody** | **Company** | **Catalog number** | **Dilution** |
| --- | --- | --- | --- |
| CD73 | Bioss, China | bs4834R | 1:500 (IF) |
| CD90 | Bioss, China | BS0778R | 1:500 (IF) |
| Nestin | Bioss, China | BS0008R | 1:500 (IF) |
| Sox2 | Bioss, China | BS0523R | 1:500 (IF) |
| Fibronectin-1 | Beyotime, China | AF6912 | 1:500 (IF); 1:2000 (WB) |
| α-SMA | Bioss, China | BSM33188m | 1:500 (IF); 1:2000 (WB) |
| Collagen I | Bioss, China | BS-10423R | 1:500 (IF); 1:2000 (WB) |
| Collagen Ⅲ | Bioss, China | BS-0549R | 1:500 (IF); 1:2000 (WB) |
| CD163 | Bioss, China | BS2527R | 1:500 (IF, FCM); 1:2000 (WB) |
| CD11b | Beyotime, China | AF6396 | 1:500 (IF); 1:2000 (WB) |
| F4/80 | Bioss, China | BS11182R | 1:500 (IF); 1:2000 (WB) |
| CCL7 | Solarbio, China | K106780P | 1:500 (IF); 1:2000 (WB) |
| GAPDH | Bioss, China | BS0755R | 1:2000 (WB) |

**Table S2 Primers**

| **Gene** | **F-Primer** | **P-Primer** |
| --- | --- | --- |
| Collagen I | GTGAACGTGGTGAAGTTGGC | TCACCACGACTTCCAACAGG |
| Collagen III | AGCCACCTTGGTCAGTCCA | GTGTAGAAGGCTGTGGGCAT |
| α-SMA | GTACCACCATGTACCCAGGC | GCTGGAAGGTAGACAGCGAA |
| Fibronectin | CCAGAGGAGGCACAAGGTTC | TGGGAAACCGTGTAAGGGTC |
| CD163 | GAGACACACGGAGCCATCAA | CGTTAGTGACAGCAGAGGCA |
| F4/80 | TCTGGGGAGCTTACGATGGA | GAATCCCGCAATGATGGCAC |
| CD11b | ATCATAGGCGCCCACTTCTC | TCTGAGCAGCAGAAGATGCC |
| CCL7 | CCATCAGAAGTGGGTCGAGG | ACCATTCCTTAGGCGTGACC |
| GAPDH | CACTGCCACCCAGAAGACTG | AGATCCACGACGGACACATT |


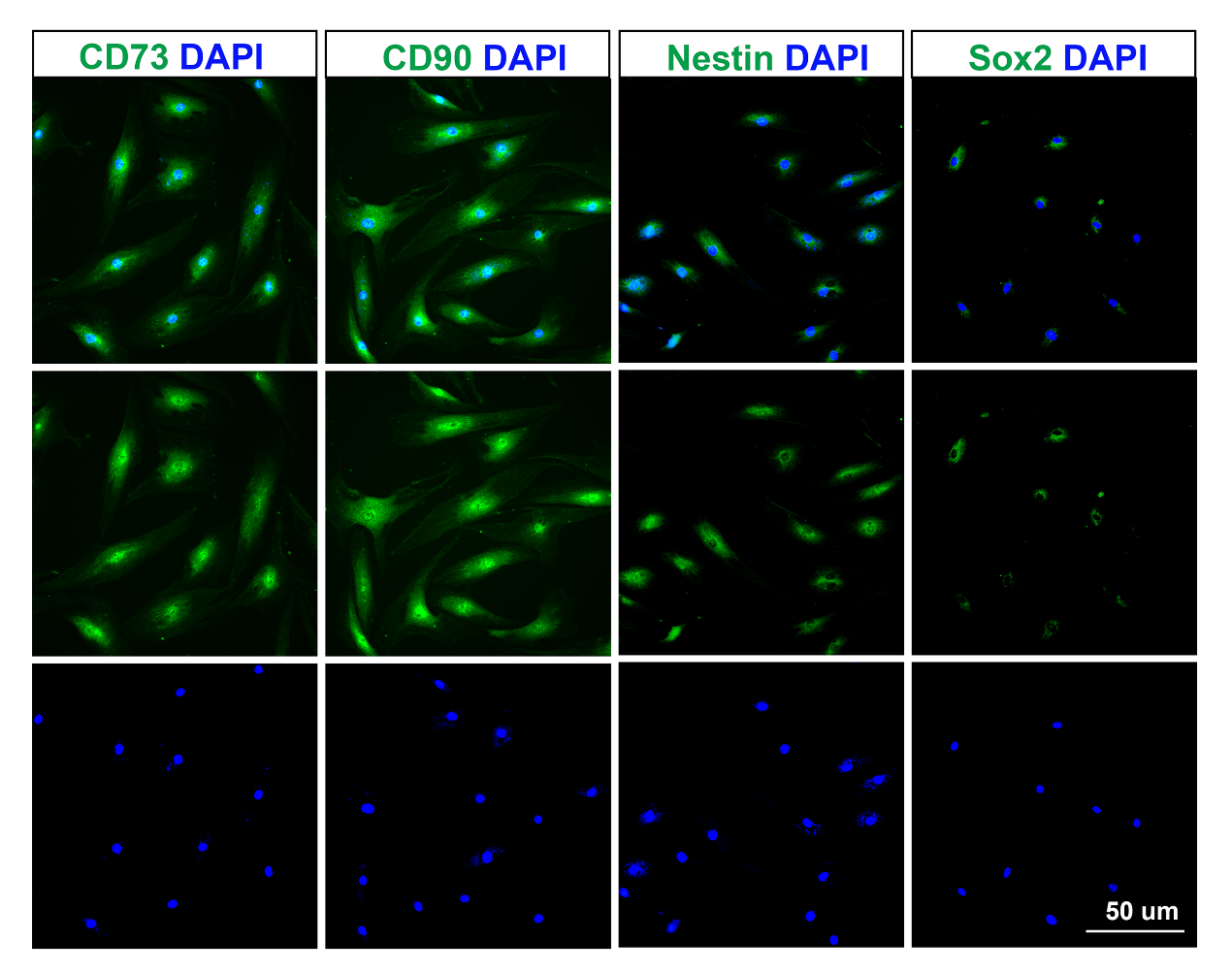


**Fig. S1 IF staining shows expression of CD73, CD90, Nestin and Sox2 in the cells (AnSCs) isolated from the initial antler blastema; scale bar = 50μm.**


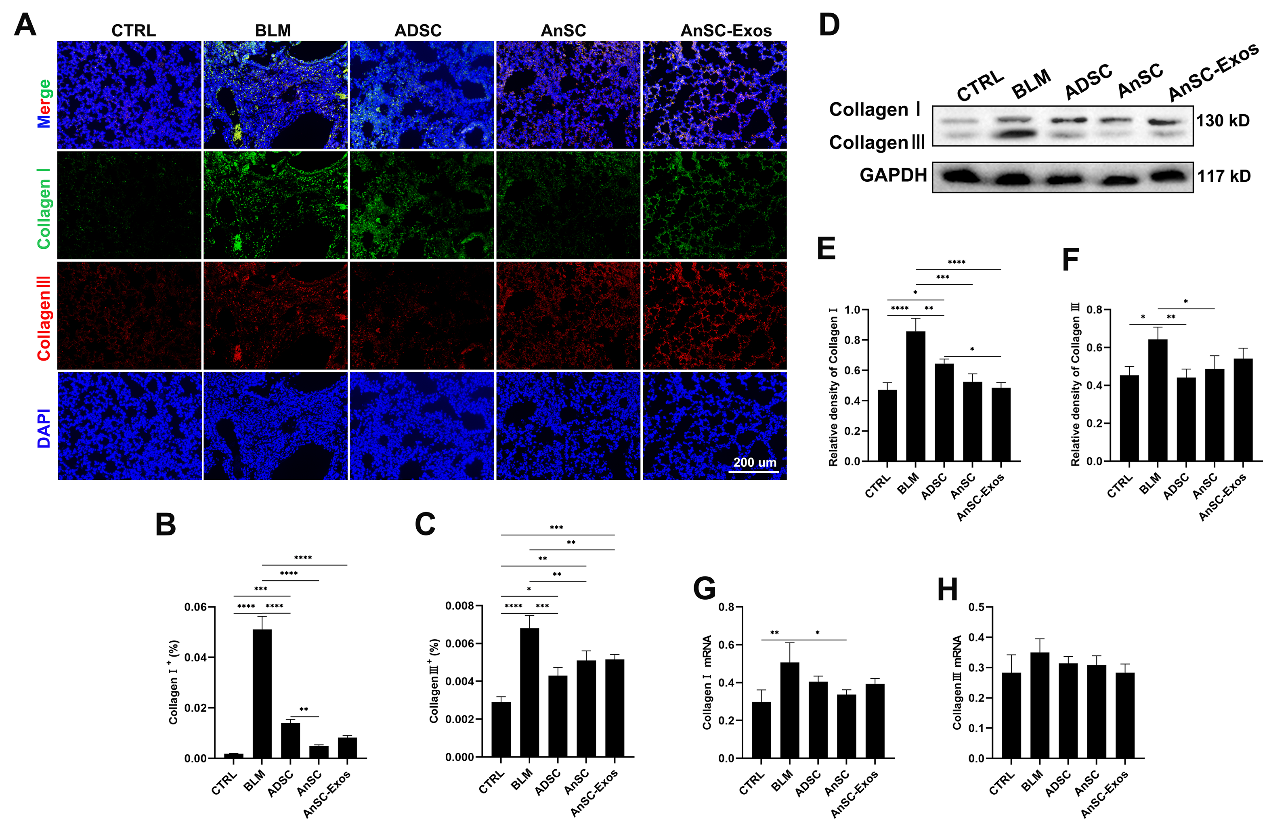


**Fig. S2 Inhibition of collagen deposition by AnSCs and AnSC-Exos in the lung tissue of BLM-induced mice. A** IF stainings of collagen I and collagen III; scale bar = 200μm. **B, C** Percentages of collagen I^+^ cells and collagen III^+^ cells, respectively. **D-F** Western blot bands and the relative intensities of collagen I and collagen III, respectively. **G, H** Relative mRNA levels of collagen I and collagen III, respectively. Value: Mean ± SEM; *p<0.05, **p<0.01, ***p<0.001, ****p<0.0001 as indicated by Student’s t test; n=3.


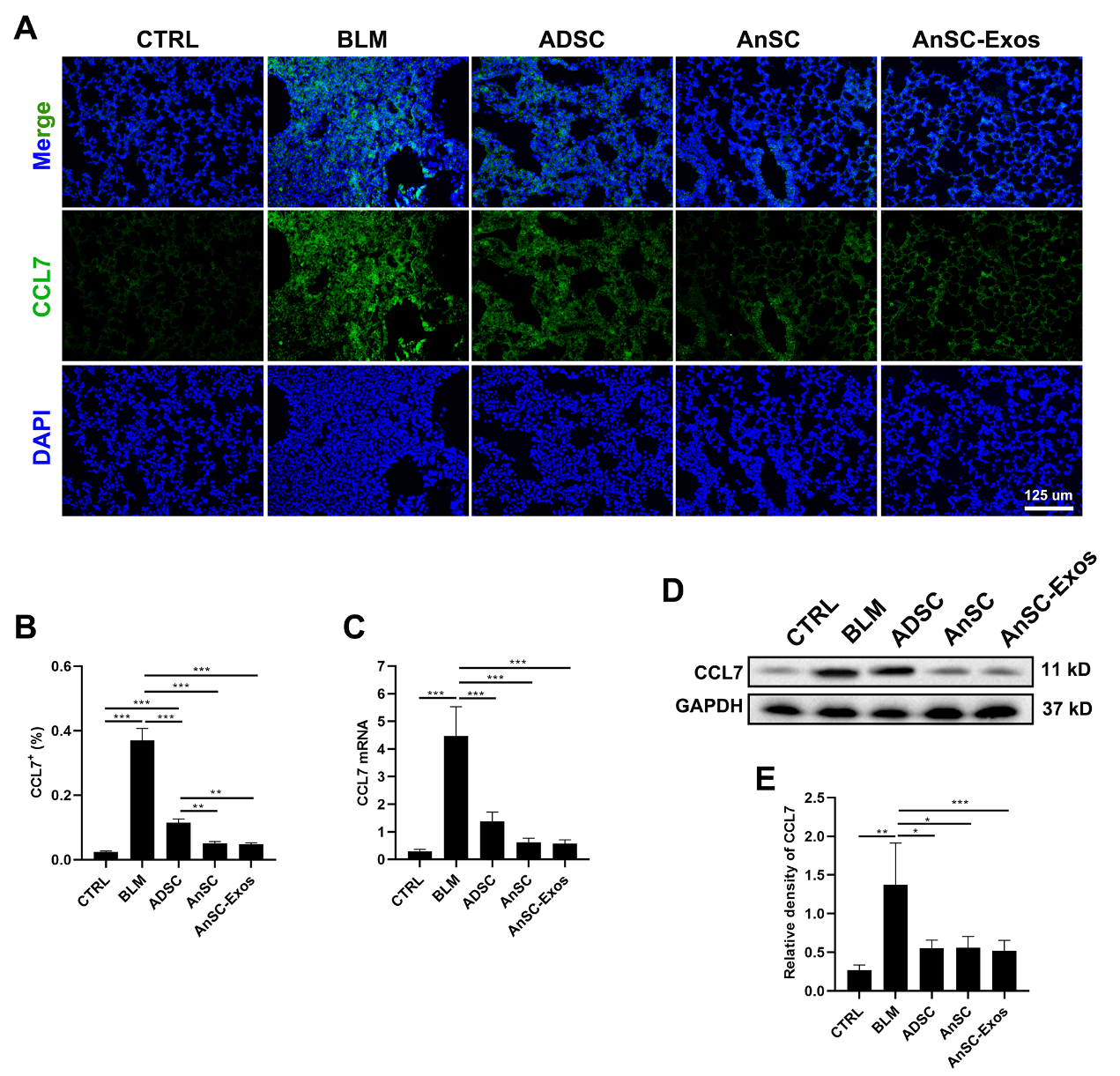


**Fig. S3 Inhibition of CCL7 expression by AnSCs and AnSC-Exos in the lung tissue of BLM-induced mice. A** IF staining of CCL7; scale bar = 125μm. **B** Percentages of CCL7^+^ cells. **C** Relative mRNA level of CCL7. **D, E** Western blot bands and the relative intensities of CCL7, respectively. Note that AnSCs and AnSC-Exos effectively inhibited CCL7 expression in the lung tissue of the model mice. Value: Mean ± SEM; *p<0.05, **p<0.01, ***p<0.001 as indicated by Student’s t test; n=3.


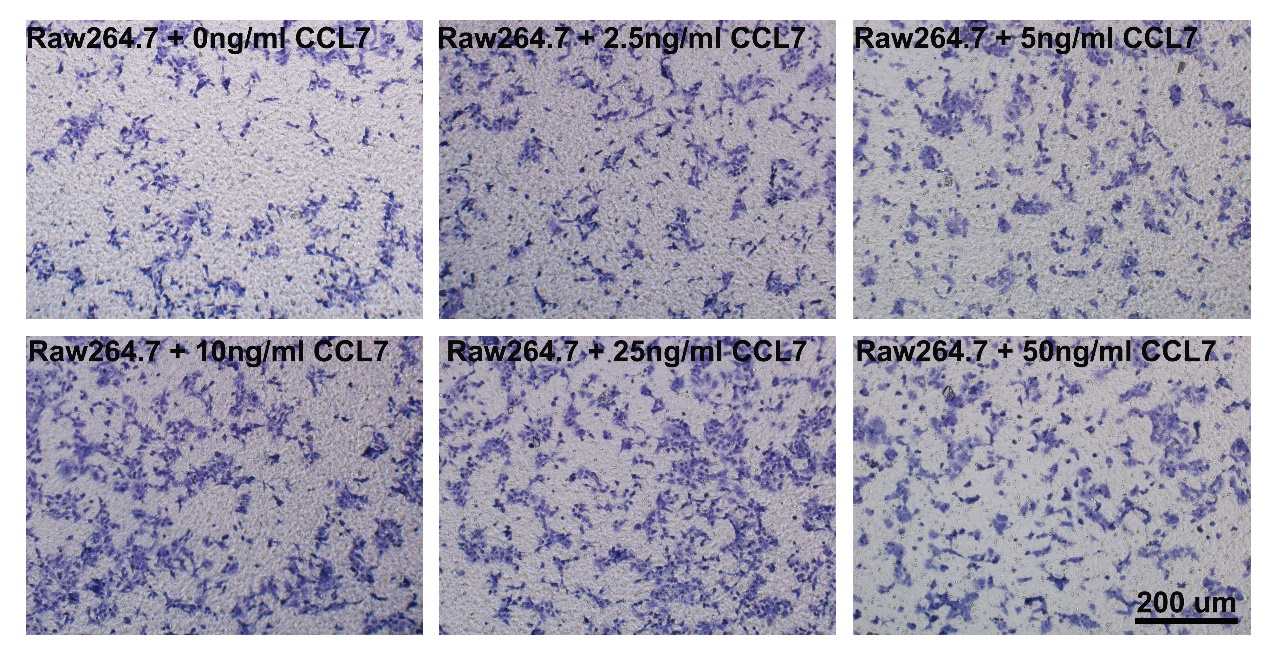


**Fig. S****4** **Effects of CCL7 on macrophage migration *in vitro*.** Raw264.7 cells were cultured in the inserts, and different concentrations of CCL7 (0, 2.5, 5, 10, 25 and 50 ng/ml) were added to the culture medium in the wells. The migrated Raw264.7 cells were stained with crystal violet; scale bar = 200μm. Note that CCL7 significantly induced the migration of macrophages in a dose-dependent manner.


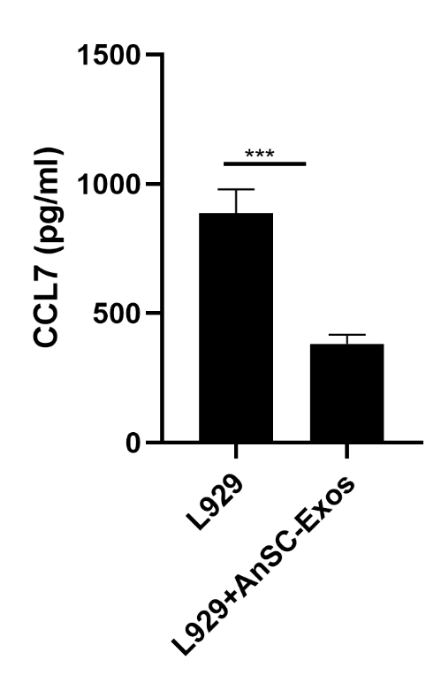


**Fig. S5 Inhibitory effects of AnSC-Exos on CCL7 expression of L929 cells *in vitro*.** Value: Mean ± SEM; ***p< 0.001 as indicated by Student’s t test; n=3.


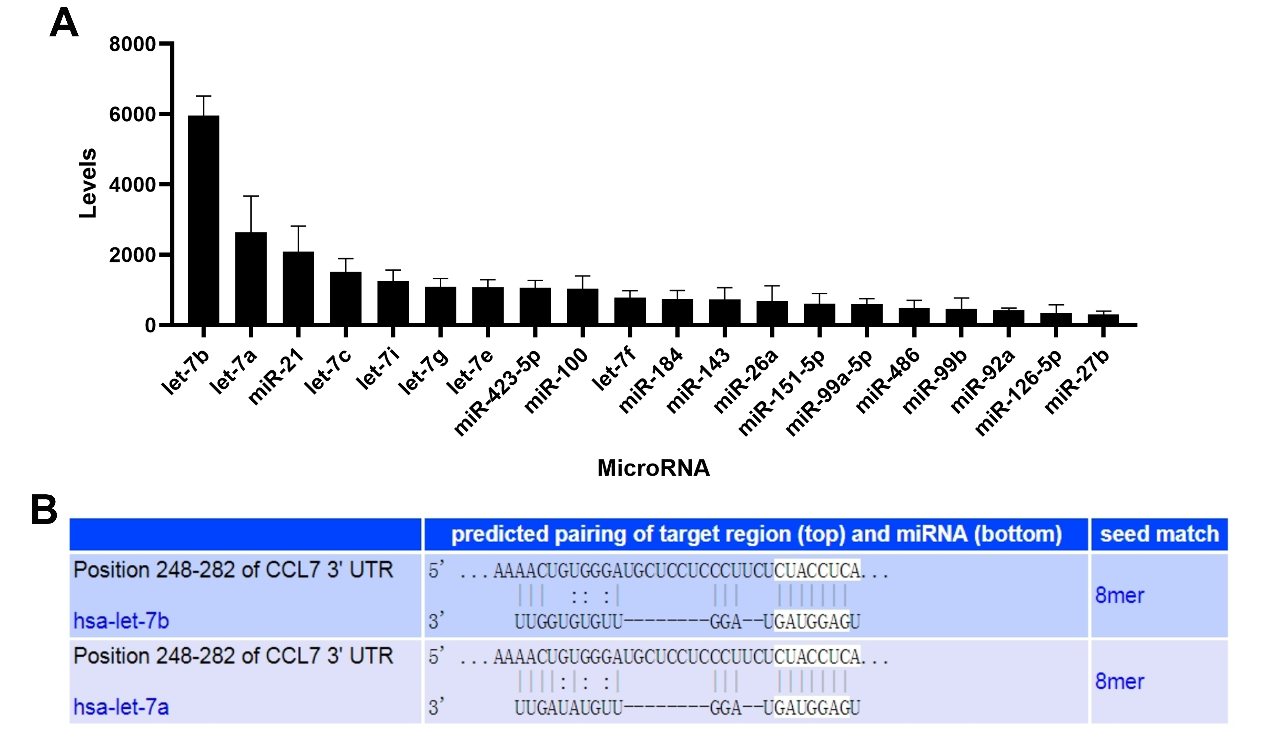


**Fig. S6** **AnSC-Exos-specific miRNAs and their predicted targets. A** Top 20 miRNAs in AnSC-Exos; Value: Mean ± SEM; n=3. **B** List of predicted binding sites for let-7b/a and their target CCL7.


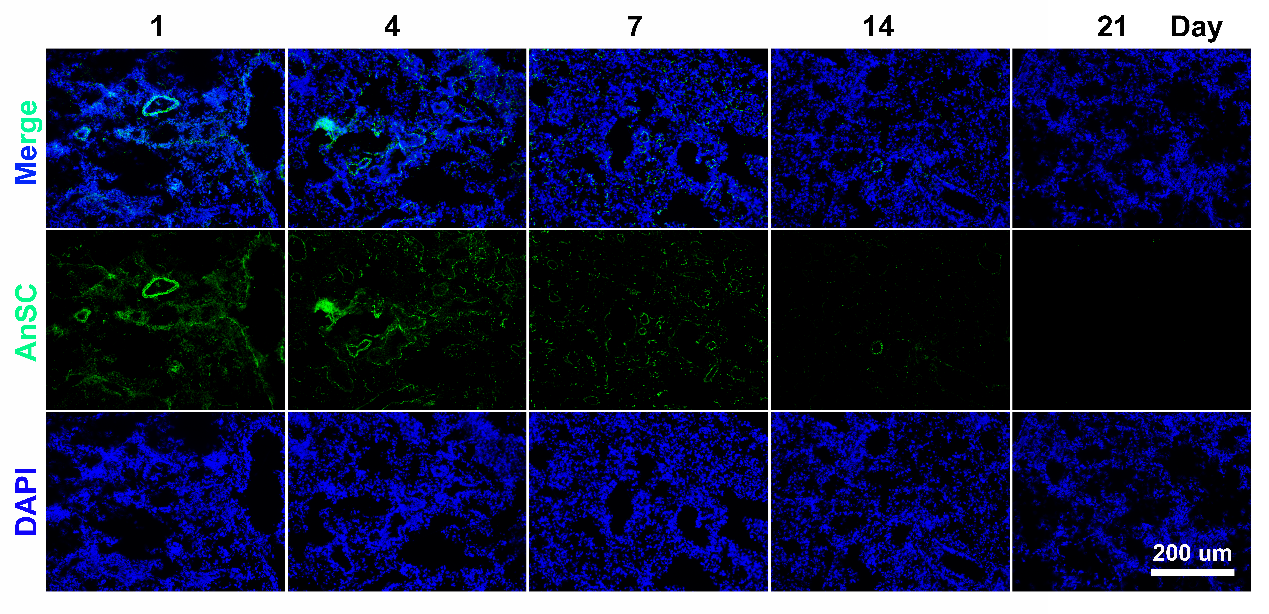


**Fig. S7 Lineage tracing of the injected AnSC cells in the lung tissue of BLM-induced mice.** CFDA-SE-labeled AnSCs were injected into the BLM-induced mice via tail veins. Lineage tracing of the AnSCs in the lung tissue was performed on days 1, 4, 7, 14 and 21 after cell injection; scale bar = 200μm. Note that AnSCs gradually decreased in number and totally disappeared on day 21.
