# Supplementary material for: Antler stem cell exosomes alleviate pulmonary fibrosis via inhibiting recruitment of monocyte macrophage, rather than polarization of M2 macrophages in mice
Source: Cell Death Discov. 2023 Sep 28;9:359. doi: 10.1038/s41420-023-01659-9 (PMC10539297; doi:10.1038/s41420-023-01659-9)
Supplement: Supplementary file 2 — Original Data File [file 41420_2023_1659_MOESM2_ESM.pdf]

Figure 3D

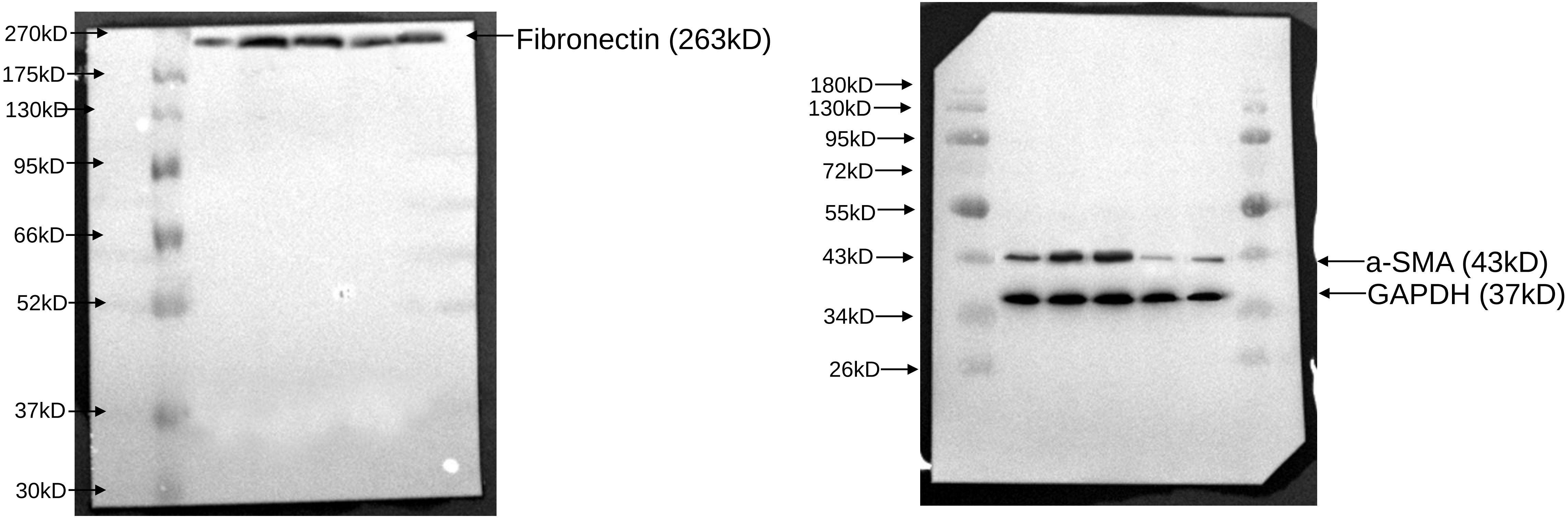
270kD
175kD
130kD
95kD
66kD
52kD
37kD
30kD
Fibronectin (263kD)
180kD
130kD
95kD
72kD
55kD
43kD
34kD
26kD
a-SMA (43kD)
GAPDH (37kD)

Figure 4D

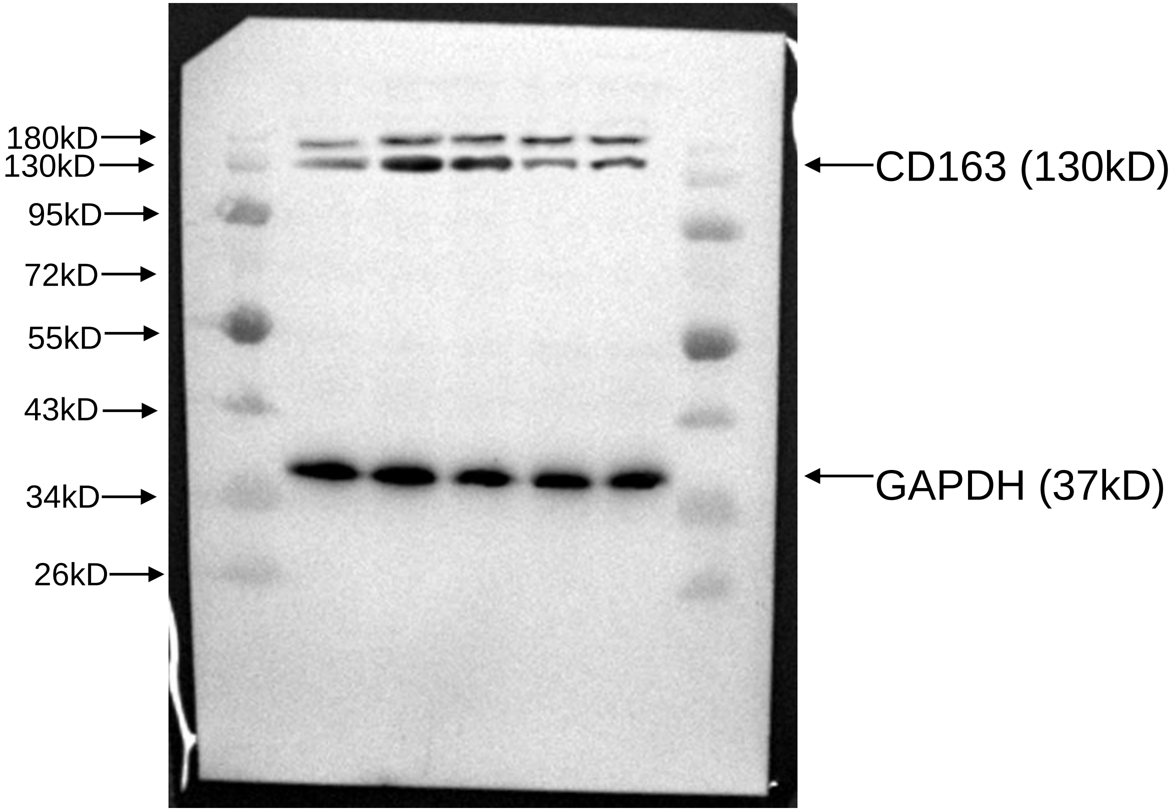

Figure 4H

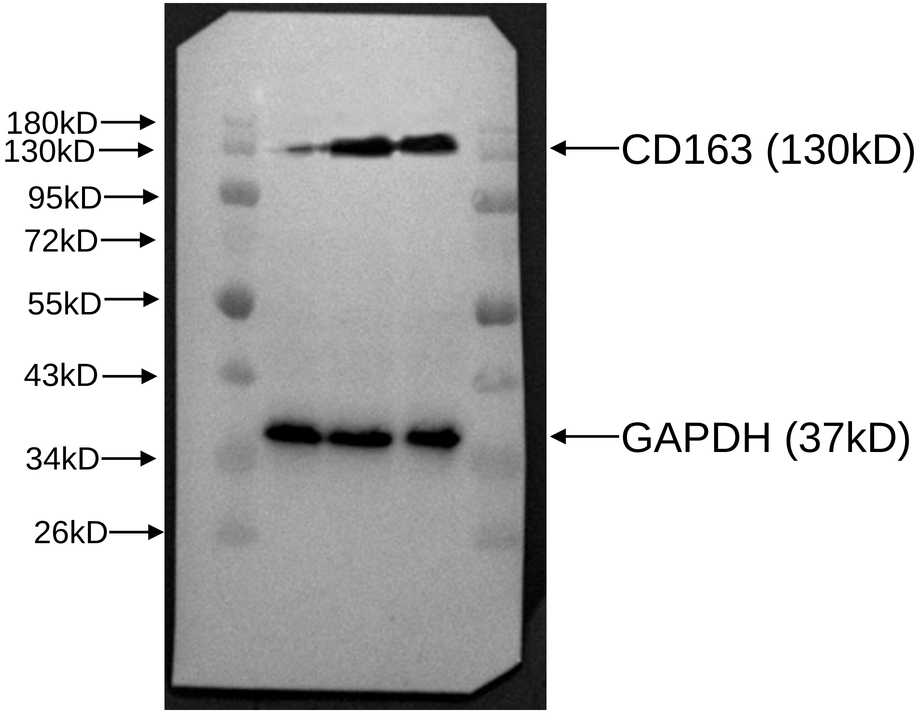

Figure 5D

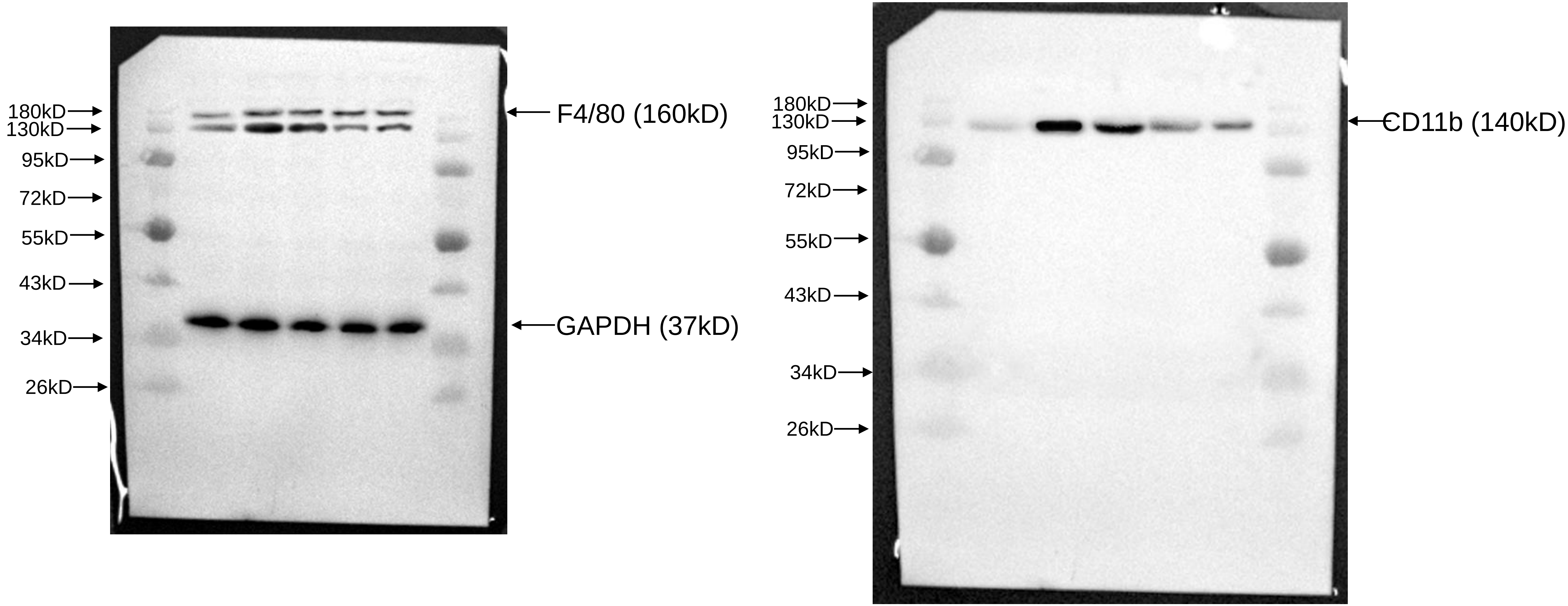
180kD
130kD
95kD
72kD
55kD
43kD
34kD
26kD
F4/80 (160kD)
GAPDH (37kD)
180kD
130kD
95kD
72kD
55kD
43kD
34kD
26kD
CD11b (140kD)

Figure 6D

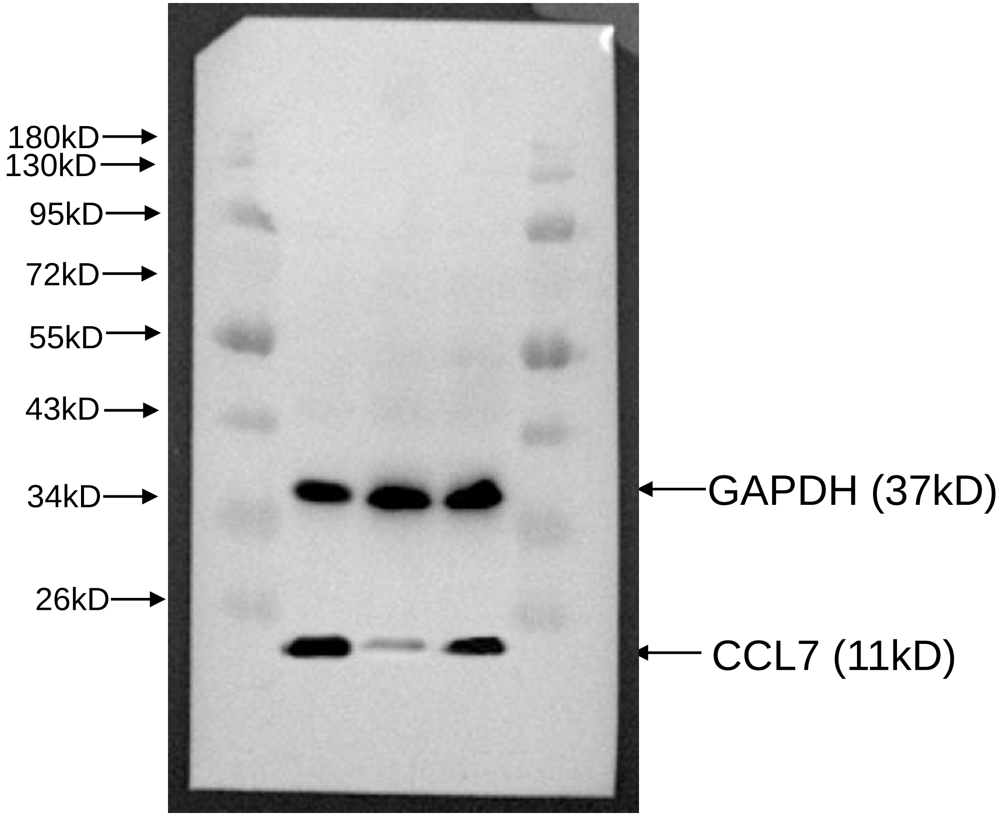

Figure 7D

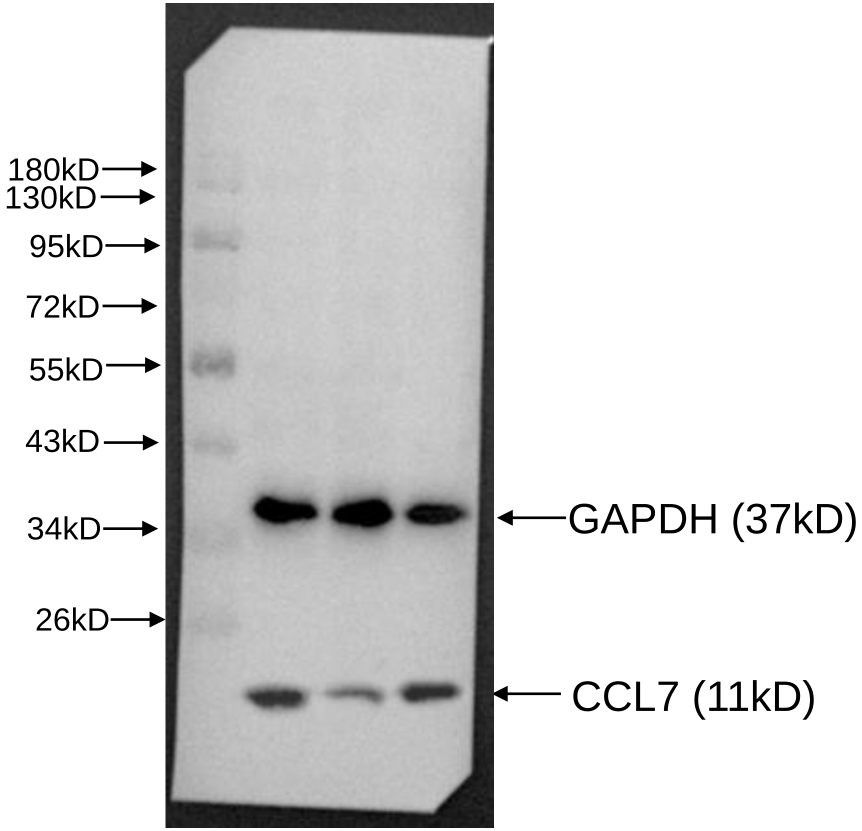

Figure S2D

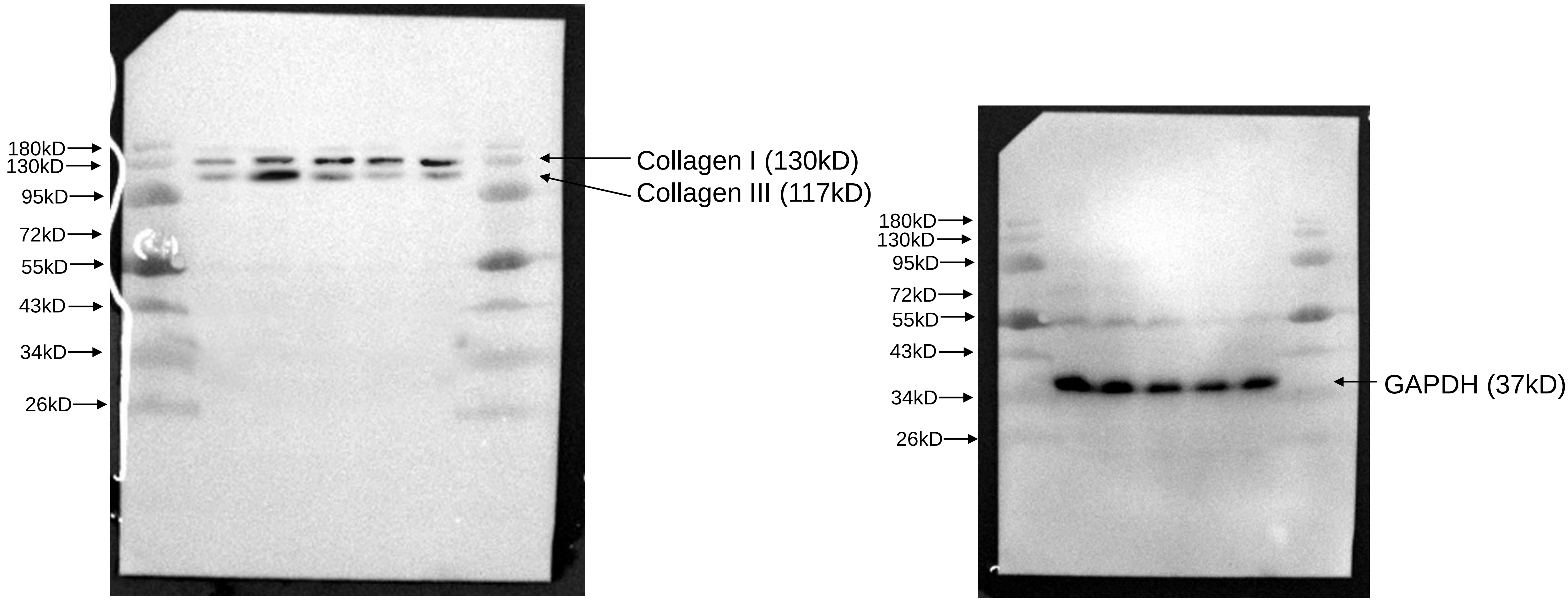

180kD
130kD
95kD
72kD
55kD
43kD
34kD
26kD
Collagen I (130kD)
Collagen III (117kD)
180kD
130kD
95kD
72kD
55kD
43kD
34kD
26kD
GAPDH (37kD)

Figure S3D

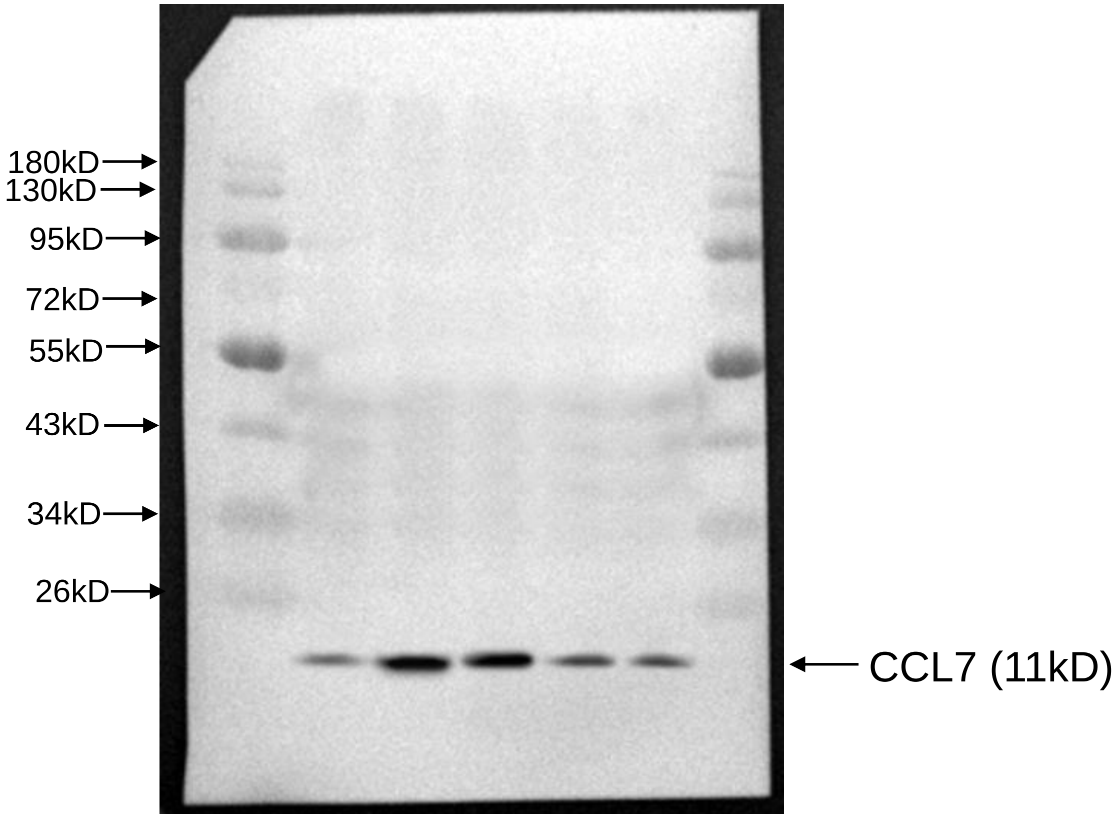
180kD
130kD
95kD
72kD
55kD
43kD
34kD
26kD
CCL7 (11kD)

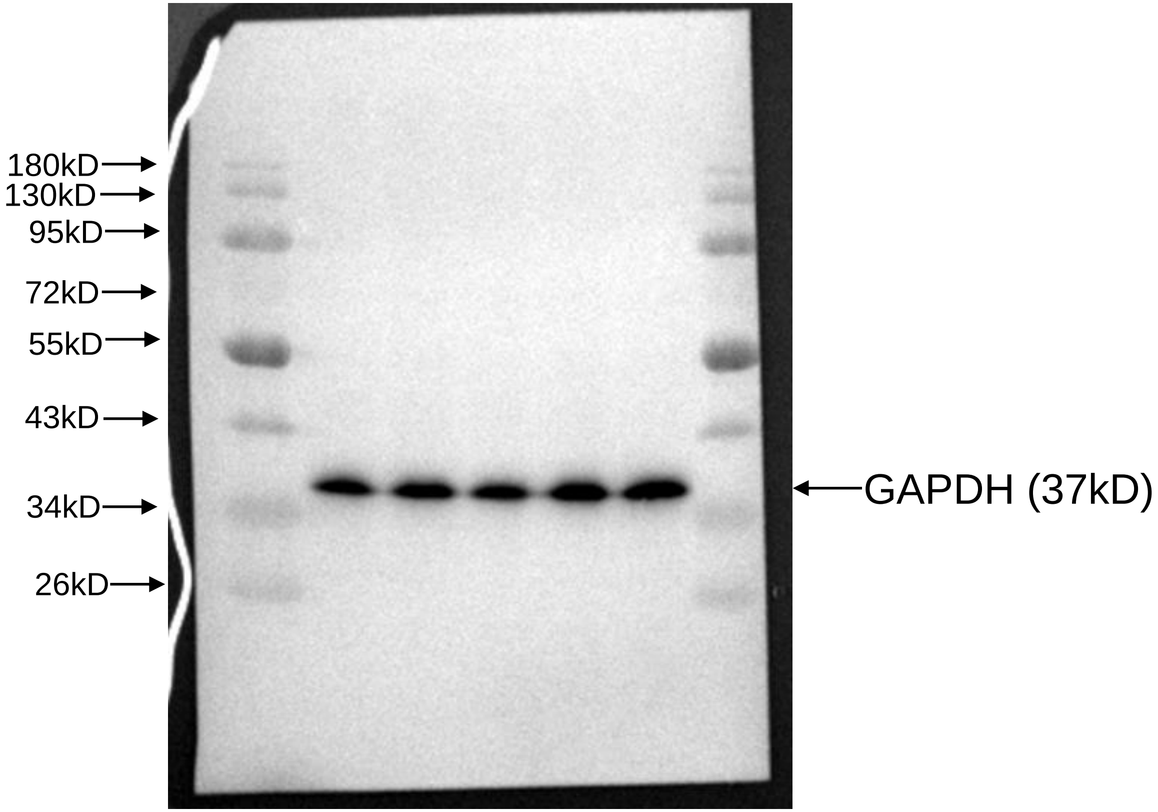
180kD
130kD
95kD
72kD
55kD
43kD
34kD
26kD
GAPDH (37kD)
